# Medical students’ coping with stress and its predictors: a cross-sectional study

**DOI:** 10.5116/ijme.63de.3840

**Published:** 2023-02-28

**Authors:** Julia Cummerow, Katrin Obst, Edgar Voltmer, Thomas Kötter

**Affiliations:** 1 Institute of Family Medicine, University Medical Centre Schleswig-Holstein, Campus Lübeck, Lübeck, Germany; 2 Institute of Social Medicine and Epidemiology, University Medical Centre Schleswig-Holstein, Campus Lübeck, Lübeck, Germany

**Keywords:** Coping skills, health promotion, medical school, medical students

## Abstract

**Objectives:**

To analyse stress coping styles of medical students at different time points of medical education and to identify predictors of functional coping.

**Methods:**

A cross-sectional study was conducted among medical students (N = 497, 361 women and 136 men) before year one (n = 141), after year one (n = 135) and after year five (n = 220). Students answered the Brief Coping Orientation to Problems Experienced Inventory, the Work-Related Behaviour and Experience Patterns, the Perceived Medical School Stress Instrument and the Maslach Burnout Inventory. Multiple regression was used to examine factors associated with functional coping.

**Results:**

Single factor ANOVA indicated a significant difference for functional coping between the time points (F _(2, 494)_ = 9.52, p < .01), with fifth-year students scoring significantly higher than students before or after year one. There was a significant difference in dysfunctional coping (F _(2, 494)_ = 12.37, p < .01), with students before year one and after year five scoring higher than those after year one. Efficacy (β = 0.15, t _(213)_ = 4.66, p < .01), emotional distancing (β = 0.04, t _(213)_ = 3.50, p < .01) and satisfaction with life (β = 0.06, t _(213)_ = 4.87, p < .01) were positive predictors of functional coping.

**Conclusions:**

Scores for both functional and dysfunctional coping vary during medical education. The reasons for low coping scores after year one require further explanation. These findings represent a starting point for investigations into how to promote functional coping during early medical education.

## Introduction

Internationally, doctors have a high stress-related risk of developing burnout symptoms,^1^^-^^3^ which affect their personal well-being and increase the occurrence of medical error.^3^^,^^4^ Already at the beginning of professional life, doctors must deal with multiple work demands while they perceive themselves as insufficiently educated for the job.^5^ Nevertheless, high stress levels are not a phenomenon unique to physicians. It has long been known that medical training places students under a high level of stress from the very beginning, a phenomenon that also puts them at increased risk of mental health disorders.^6^^,^^7^ Work and behaviour patterns with a high risk of burnout increase in the first years of medical education,^8^ whereas life satisfaction deteriorates.^9^ Examples of study-related stressors that may affect mental and physical health are high workload, curricular demands, ethical dilemmas, confrontation with suffering and dying among patients and financial burdens. Consequences, among others, can exert negative effects on academic performance, increasing cynicism; decreasing empathy; and promoting academic dishonesty, harmful substance use and even suicidal thoughts and plans.^7^ Thus, measures to reduce stress and improve medical students’ health – if possible, even with lasting effects later in their careers – have become an increasing subject of research.^10^^,^^11^ Nevertheless, appropriate starting points that provide long-term effects still need to be identified.

Among other factors, the way medical students cope with stress is important in this context. According to the transactional model of stress and coping by Lazarus and Folkman,^12^ coping is a cognitive and behavioural process to deal with external, internal or combined stressors that are assessed as burdensome or overextend the internal resources. Dunn and colleagues^13^ described the ‘coping reservoir’ model, which is filled with personal traits, temperament and the coping reserve. Negative influences such as stress, internal conflicts and time and energy demands empty this reserve, while positive influences such as psychosocial support, social/healthy activities, mentorship and intellectual stimulation fill it up. These changes have corresponding effects on the students’ well-being. Researchers have also reported on the effects of specific coping strategies. However, it should be noted that the nomenclature of coping strategies differs depending on the study and the instrument used. A high perceived stress load can lead to increased use of unfavourable coping strategies,^6^ which are, in turn, associated with a higher perception of stress.^14^ Active coping and positive thinking, on the other hand, are associated with lower perceived stress levels.^15^

The association between coping strategies and mental^16^^,^^17^ and physical health^16^ has long been known. More precisely, mental disorders and student burnout are associated with the pronounced use of unfavourable coping strategies like self-blaming; denial; substance abuse;^18^ behavioural disengagement;^19^ and insufficient support from family, friends and fellow students.^20^ In contrast, approach-oriented or active coping strategies and positive thinking reduce the risk of burnout and depression.^15^^,^^20^ Furthermore, active coping is positively correlated with resilience.^21^ Nevertheless, little is known about factors to improve the use of these coping strategies.

Due to their associations with coping, it is also remarkable that mental and general health decline in the first year of medical education^22^^,^^23^ and improve afterwards, but without returning to the initial levels.^22^ However, data on coping in different years of medical school are not as clear. A recent Canadian study reported greater use of denial by third-year students compared with those in the other years of study.^24^ In a Malaysian study, first-year students had significantly higher scores for task-oriented coping than those in their third year, whereas there were no differences in avoidant and emotion-oriented coping.^25^ Comparison between undergraduate and graduate-entry students with another previous degree showed higher use of active coping, positive reframing and substance use for the latter, and higher scores of religious coping for the former.^26^

Our first objective in the present study was to evaluate whether and how coping styles vary in different years of medical education. To this end, we conducted a detailed analysis of coping styles at three different points in medical school. Furthermore, the aforementioned association of health and coping styles leads to the consideration of coping is a possible starting point for health-promoting measures. Therefore, our second objective was to identify factors that increase the use of functional coping strategies. Thus, we analysed the association between functional coping and study-related health and behaviour patterns and perceived study-related stress. Considering the impact of coping strategies on student burnout, we also analysed the reverse path – that is, the influence of the three burnout dimensions on coping behaviour.

## Methods

### Study design and participants

We gathered the cross-sectional data presented in this report as part of an ongoing single-centre study on student health (Lübeck University Students Trial [LUST]). For further information, see Kötter and colleagues.^27^ We analysed data from medical students at three different time points: at the beginning of their education (t0) in 2017, at the end of their first year (t1) in 2016 and at the end of their fifth year (t2) (in 2016 and 2017).

The study was approved by the Ethics Committee of the University of Lübeck (file reference 11-010). Informed consent was obtained from all the participants. We performed the analysis with anonymised data.

### Measures

Every questionnaire consisted of sociodemographic data and a number of psychometric instruments. For the present study, all of the participants answered the Brief Coping Orientation to Problems Experienced (COPE) Inventory. For the participants at the end of their fifth year, we also analysed the ‘Arbeitsbezogenes Verhaltens- und Erlebensmuster’ (AVEM-questionnaire) [Work-Related Behaviour and Experience Patterns], the Perceived Medical School Stress Instrument (PMSS) and the Maslach Burnout Inventory (MBI) to identify predictors of functional coping.

### Brief COPE Inventory

The Brief COPE Inventory^28^ is the short form of the COPE Inventory.^29^ It comprises 14 coping strategies: acceptance, use of emotional support, humour, positive reframing, active coping, use of instrumental support, planning, behavioural disengagement, denial, self-distraction, self-blame, venting, religion, and substance use. Each coping strategy is determined by two items, with response options presented as 4-point Likert scales from 1 (I haven’t been doing this at all) to 4 (I’ve been doing this a lot). Cronbach’s Alpha for each scale is ≥ .50. We used the German version^30^ of the situational questionnaire referring to a specific stressful situation in the past.^28^^,^^29^ We used a classification of functional and dysfunctional coping strategies similar to the one described by Frost and Mierke^31^ in a survey with a large number of students (N = 1014) of several German universities. Like them, we used the means for further analysis. However, we excluded religion and venting. While venting may be considered functional for a person in mourning, it may impair healthy actions in other situations due to a dysfunctional focus on the stressor.^29^ As we did not estimate the personal context, it was not possible to provide an individual assignment to a higher-order dimension. In addition, religion is a coping strategy that cannot simply be classified as functional or dysfunctional.

While some researchers have reported a positive association between religion and psychological health,^18^ others have shown an association between religion and higher stress scores.^15^ The authors of a recent scoping review concluded that positive religious coping has an adaptive effect, while negative religious coping has a maladaptive effect.^32^ Without further contextual information, we decided to exclude this strategy from further analysis as well. Thus, in the present study functional coping consisted of the variables acceptance, use of emotional support, humour, positive reframing, active coping, use of instrumental support, and planning, whereas dysfunctional coping comprised behavioural disengagement, denial, self-distraction, self-blame, and substance use.

### AVEM-questionnaire

The AVEM-questionnaire^33^ is a validated instrument to detect self-reported personal experiences and ways of coping with work-related stress. We used the abbreviated, study-related version comprising 44 items, which has been widely used in studies on student health in Germany.^8^^,^^31^ The instrument consists of 11 scales each comprising four items, with answers given on a 5-point Likert scale from 1 (I strongly disagree) to 5 (I strongly agree). The scales are: subjective significance of work, career ambition, tendency to exert, striving for perfection, emotional distancing, resignation tendencies, offensive coping with problems, balance and mental stability, satisfaction with work, satisfaction with life, and experience of social support. These scales have a high reliability with Cronbach’s Alpha between .78 and .87.^33^ Due to their similarity to coping, we excluded the AVEM dimensions offensive coping with problems and experience of social support from further analyses.

### PMSS

The PMSS was developed by Vitaliano and colleagues^34^ and has been widely used to capture the self-rated stress load of medical students.^6^^,^^35^ We used the translated and validated German version (PMSS-D),^36^ which consists of 13 items. The reliability is reported as good (Cronbach’s Alpha = .81). Answers are given on a 5-point Likert scale from 1 (I strongly disagree) to 5 (I strongly agree). A high total score (range 13-65) represents a high stress load.^36^ Because we investigated study-specific stressors, we did not use the instrument for the students at the beginning of their studies, because they had not yet experienced study-related stress.

### MBI

The MBI is an instrument used to determine the severity of burnout syndrome.^37^ The version we used (MBI-SS-GV) has been modified for use in students by Schaufeli and colleagues^38^ and translated into German and validated by Gumz and colleagues,^39^ who also reported high reliability of the subscales (Cronbach’s Alpha between .81 and .86). This questionnaire consists of 15 items with response options based on a 7-point Likert scale from 0 (never) to 6 (daily). The 15 items are divided into the scales emotional exhaustion (five items), cynicism (four items) and efficacy (six items). The evaluation is based on mean values. High emotional exhaustion and cynicism scores and low efficacy scores indicate the presence of burnout syndrome.^39^ The subscales (each with a range of 0-6) are evaluated separately; a total score is not obtained.^37^ Due to the different compositions of the questionnaires, data on the MBI were only available for the t2 respondents.

### Data collection and study setting

The paper-based baseline survey (t0) was taken in October 2017 during the medical pre-course week (prior to the start of medical courses). The web-based surveys after the first year (t1) and after the fifth year (t2) were conducted at the end of the summer semesters in June 2016 (t1 and t2) and 2017 (t2), respectively, using web surveys. There were no exclusion criteria. The study size was predefined by the size of the medical classes at the University of Lübeck (185 students per class). To reduce bias due to non-response, posters, leaflets, information via well-attended lectures, emails and Facebook were used to encourage participation. Each participant was offered a 5€ voucher for a local bakery or bookstore for each survey. The study was conducted at the University of Lübeck, Germany, a public university with a focus on medicine and life sciences.

### Data analysis

We used IBM SPSS Statistics for Windows Version 22.0 (released in 2013, IBM Corp., Armonk, NY, USA) for data analysis. Where possible, we substituted missing values by following the rules provided in the handbooks for the instruments. We excluded any remaining incomplete data sets. We used two-tailed statistical tests with a level of significance of .05. We used the unequal variance t-test to compare the means between two independent samples. We analysed the mean differences between t0, t1 and t2 by using single-factor analysis of variance (ANOVA) and a Tamhane T2 post hoc test appropriate for unequal variances.^40^ We calculated the effect sizes of mean differences (Cohen’s d) by using an online tool^41^ (small effect 0.2, medium effect 0.5 and large effect 0.8).^42^ We analysed categorial data with the chi-square test and report the results as percentages. We used Pearson’s correlation to examine associations between the coping strategies and the scales of the other instruments. We analysed the predictors of functional coping by multiple regression after checking for multicollinearity. We chose an explorative approach. To remove variables without significant influence on the model quality, after forced input of all potential predictors we chose a stepwise backwards elimination.

## Results

### Participants

After excluding incomplete data sets, we analysed cross-sectional data from 497 medical students before starting medical school (t0), after their first year (t1) and after their fifth year (t2). The data for the t0 and the t1 surveys are from the same class, while the data for the t2 survey are from two classes (Table 1). These two classes did not differ significantly in terms of age (t _(209.30)_ = 0.33, p = .74) and gender (χ² _(1, N = 220)_ = 1.81, p = .18), and thus we combined them as t2 for further investigation. The mean age of the participants in years was 23.34 (SD = 3.58). The proportion of female participants was slightly higher at t2 (76%) compared with t0 and t1 (70% at each time point). With a presumed number of 185 students per class, the response rates were 76% at t0, 74% at t1 and 59% at t2.

**Table 1:** Participant demographics

Participants (Year of Survey)	n	Age (years)^*^		Male	Female
		M	SD	n (%)	n (%)
t0 (2017)	141	21.42	3.39	43 (30)	98 (70)
t1 (2016)	136	22.62	4.05	41 (30)	95 (70)
t2 (2016/2017)	220	25.01	2.46	52 (24)	168 (76)
t2a (2016)	109	25.06	2.66	30 (28)	79 (72)
t2b (2017)	111	24.95	2.26	22 (20)	89 (80)
Total	497	23.34	3.58	136 (27)	361 (73)

*Seven participants did not provide their age. t0 = beginning of medical school, t1 = after the first year of medical school, t2 = after the fifth year of medical school

Coping at t0, t1 and t2

At each time point, medical students scored higher for functional coping than for dysfunctional coping (Table 2). Single-factor ANOVA revealed significant differences between the years of medical school for functional coping (F_(__2, 494)_ = 9.52, p < .01) and for dysfunctional coping (F_(2, 494)_ = 12.37, p < .01). The Tamhane T2 post hoc test (Table 3) indicated a significant difference between the scores of functional coping of students before beginning medical school (M = 2.74, p = .02) and after their first year of study (M = 2.66, p < .01) compared with after their fifth year of study (M = 2.88). There was no significant difference in the functional coping scores for students before beginning medical school and after their first year of medical education (Table 3). The Tamhane T2 post hoc test revealed a significant difference between the dysfunctional coping scores of students before beginning medical school (M = 1.77, p < .01) and after their fifth year of study (M = 1.83, p < .01) compared with after their first year of study (M = 1.66). The difference between t1 and t2 for dysfunctional coping had a medium effect size (Cohen’s d = 0.54). There was no significant difference in the dysfunctional coping scores for students before beginning medical school and after their fifth year of study (Table 3).

**Table 2:** Functional and dysfunctional coping at t0, t1 and t2

Coping	t	n	M	SD
Functional coping	t0	141	2.74	0.47
	t1	136	2.66	0.56
	t2	220	2.88	0.46
Dysfunctional coping	t0	141	1.77	0.33
	t1	136	1.66	0.33
	t2	220	1.83	0.31

Note: t0 = beginning of medical school, t1 = after the first year of medical school, t2 = after the fifth year of medical school

**Table 3:** Single-factor analysis of variance and Tamhane T2 post hoc test results and effect sizes of functional and dysfunctional coping

Coping	df	F	p	Difference between group scores		p	Cohen’s d
Functional coping	2, 494	9.25	< .01	t0-t1	0.08	.46	0.16
				t2-t0	0.14	.02	0.30
				t2-t1	0.23	< .01	0.44
Dysfunctional coping	2, 494	12.37	< .01	t0-t1	0.12	< .01	0.33
				t2-t0	0.06	.30	0.19
				t2-t1	0.17	< .01	0.54

Note: nt0 = 141, nt1 = 136 and nt2 = 220. t0 = beginning of medical school, t1 = after the first year of medical school, t2 = after the fifth year of medical school

### Gender-specific coping

In the total sample, there was no difference in the functional coping scores between male (M = 2.73, SD = 0.53) and female (M = 2.80, SD = 0.49) students (t _(228.44)_ = -1.45, p = .15). Moreover, there were no differences in the dysfunctional coping scores between male (M = 1.77, SD = 0.35) and female (M = 1.77, SD = 0.32) students (t _(226.79)_ = 0.11, p = .91). Comparison of the functional and dysfunctional coping scores of male and female students at each study stage also revealed no significant differences. Hence, we did not consider gender in the subsequent analysis.

### Predictors of functional coping at t2

To identify factors that foster or impede functional coping, we first assessed the correlations of functional coping with the AVEM dimensions, representing the experience of and dealing with work-related stress, with the MBI scales that measure the three dimensions of burnout, and with the perceived stress load (PMSS-score). Data for all of these variables were only available for students after their fifth year of study (t2). The following variables showed a significant correlation (positive or negative) to functional coping: the AVEM dimensions satisfaction with life (r _(492)_ = .39, p < .01), emotional distancing (r_(492) _= .25, p<.01), resignation tendencies (r _(492)_ = -.28, p < .01), balance and mental stability (r _(492)_ = .20, p < .01), satisfaction with work (r _(492)_ = .23, p < .01), subjective significance of work (r _(492)_ = -.11, p = .02) and tendency to exert (r _(492)_ = -.11, p < .01); the MBI scales cynicism (r _(214)_ = -.21, p < .01), exhaustion (r _(214)_ = -.16, p = .02) and efficacy (r _(214)_ = .38, p < .01); and the PMSS-score (r _(347)_ = -.19, p < .01). We preselected these variables as possible predictors of functional coping.

We then performed multiple regression to predict functional coping based on the PMSS-score and the AVEM dimensions and MBI scores that showed significant correlations with functional coping. Based on the test for multicollinearity, we did not have to exclude any variables. Backwards elimination (Table 4) revealed satisfaction with life (β = 0.06, t _(213)_ = 4.87, p < .01), emotional distancing (β = 0.04, t _(213)_ = 3.50, p < .01) and efficacy (β = 0.15, t _(213)_ = 4.66, p<.01) are significant positive predictors of functional coping. We found that efficacy shows the strongest correlation with functional coping. The PMSS-score did not predict functional coping (β = 0.01, t _(213)_ = 1.91, p = .06). The model explained 28% of the variance in the students’ functional coping scores (corrected R² = .28, F _(4, 213)_ = 21.68, p < .01).

**Table 4:** Predictors of functional coping (range 1–4) at t2

Variable (range)	β	SE	t	p	95% CI
Constant	0.38	0.37	1.03	.31	-0.35-1.10
Emotional distancing (4-20)	0.04	0.01	3.50	< .01	0.02-0.06
Satisfaction with life (4-20)	0.06	0.01	4.87	< .01	0.04-0.09
Efficacy (0-6)	0.15	0.03	4.66	< .01	0.08-0.21
PMSS-score (13-65)	0.01	0.00	1.91	.06	0.00-0.02

Note. n = 218 (n = 2 data sets were excluded due to missing values); F _(4, 213)_ = 21.68, p < .01; corrected R² = .28. β = regression coefficient, CI = confidence interval, PMSS = Perceived Medical School Stress Instrument, SE = standard error

## Discussion

In our cross-sectional study including medical students at three different time points of study, students after their fifth year (t2) had significantly higher functional coping than students who had not yet begun medical school (t0) and those after the first year of study (t1). In turn, dysfunctional coping was significantly higher for students who had not yet begun medical school and for those after their fifth year of study compared with those after their first year of study. A remarkable trend for both functional coping and dysfunctional coping was the lower scores of students after their first year of study compared with those who had not yet begun medical school and those after their fifth year of study.

### Coping at different time points of medical school

In accordance with other studies,^43^^,^^44^ we found predominantly higher scores for functional rather than dysfunctional coping strategies. What is striking is the lower expression of both functional and dysfunctional coping in students after their first year of medical school compared with the other investigated time points. This is reminiscent of the course of mental and physical health of medical students, which also decreases in the first year and then increases again.^22^^,^^23^ The association between some coping strategies and mental and physical health described in the Introduction could be reflected there.

On the other hand, dysfunctional strategies were only significantly different between students who had not yet begun medical school and those at the end of their first year. Factors that might increase maladaptive and even health risk coping behaviour are either very low or very high perceived autonomy (due to a U-shaped relationship) and high quantitative study-related demands.^45^ Both autonomy and workload might be perceived differently at the three time points evaluated in this study and could be of interest for further investigation. In contrast, there were no significant differences in functional coping between students who had not yet begun medical school and those at the end of their first year, but there were significant differences between both groups and fifth-year students (with the latter scoring higher). This suggests that first-year medical students, in particular, need support in training functional coping mechanisms. Furthermore, the varying demands caused by curricular features and examinations at the different time points evaluated in this study should be considered. In Lübeck, medical students complete the anatomy course after the first year. In another German medical school (Magdeburg), 50% of the medical students reported (strong) concerns about the imminent confrontation with a corpse before beginning the dissection courses, partially (39%) associated with (great) anxiety, whereas during the course most students had been more relaxed than previously expected.^46^ Thus, expectations and fears of students before beginning medical school might cause higher demands on coping, and higher anxiety-related use of dysfunctional coping, than for their counterparts after the first year. For the latter, a kind of relaxing period could have occurred, supported by the absence of major examinations after the first year. Previous research has proved that students exhibit a higher level of problem-focused or, respectively, active coping strategies and acceptance (subsumed here under functional coping) in the time before than after the exams.^44^^,^^47^ The more importance a student attaches to an exam and the more difficult it is expected to be, the greater the use of problem-focused coping.^47^ In Germany, after the fifth year of study, most students face the second part of the medical examination. Consequently, fifth-year students are likely to be under higher examination pressure and, therefore, might have higher general demands on coping than students after their first year.

### Predictors of functional coping

The prevailing coping strategies of medical students not only affect parts of their academic performance,^44^^,^^48^ but also their future success and attitude towards their profession,^49^ and their mental health as physicians.^35^ Thus, predictors of functional coping might be important for both medical students and doctors. Because the fifth-year students in our study had the highest functional coping scores, the analysis could provide important clues as to what can be learned from them in terms of supporting first-year students with significantly lower use of functional coping.

Based on our findings, one of the positive predictors of functional coping is satisfaction with life. This is in accordance with the results of Kjeldstadli and colleagues,^9^ who reported an association between stable, high life satisfaction of Norwegian medical students and greater use of problem-focused coping strategies and seeking social support rather than wishful thinking. Fostering high satisfaction with life can hardly be achieved by single measures; rather, it requires comprehensive health-promoting interventions at medical school.

Researchers have described the positive prediction of better self-rated mental health by emotional distancing.^23^ Thus, it is not surprising that it also predicts greater use of functional coping. However, some medical students are reported to face an internal conflict: they consider emotional distancing necessary for professional reactions in difficult situations but find it hard to generate a balance with empathetic reactions during patient contact.^50^ Furthermore, good emotion regulation reduces the health-risk tendency to prolong working hours.^45^ Therefore, fostering an emotional balance should be a topic of medical education and might support functional coping.

We also found that self-rated efficacy predicts functional coping and this trait should be fostered by learning and time management courses as well as manageable curricular learning content, among other approach. Gumz and colleagues^39^ showed higher efficacy scores for students of several disciplines if they had high social support. On the contrary, during the first years of medical studies in Germany, there is a decline in the experience of social support.^8^ Thus, medical schools should take responsibility to improve the social support provided to their students, not least to support their efficacy.

Perceived stress showed a significant negative correlation with functional coping. Thus, we expected the PMSS-score to be a negative predictor of functional coping. On the contrary, we found a positive association, but without significance. One explanation could be that perceived stress makes the coping response necessary only to a small extent.

### Strength and limitations

Even though we have used cross-sectional data, our study provides broad insight into the coping behaviour of medical students from the beginning to the end of the theoretical semesters. However, the use of self-report instruments carries the risk of bias due to social desirability. For example, the values for functional coping strategies might have tended to be higher and the values for dysfunctional strategies lower than the actual reality. In addition, the coping questionnaire items refer to the past, which could lead to recall bias. The response rates before the beginning and after the first year were higher than after the fifth year, which could have caused selection bias and might also be a reason for the higher proportion of female students after the fifth year compared with the other time points. The generally high proportion of females in the sample could be due to gender response bias. Indeed, a previous study found that the response rate of female students to web-based and paper-based questionnaires is higher than for male students.^51^ Nevertheless, the gender distribution in our sample at the beginning and after the first year of medical school is comparable to the national gender distribution of freshmen in German medical schools.^52^ In addition, there was no significant gender difference for functional and dysfunctional coping. A further limitation is given by the single-centre study design, affecting in particular the generalisability of the results. Due to data availability, we could only evaluate predictors of functional coping for fifth-year students. Because their functional coping response differed from those who had not yet begun medical school and those after their first year, there might also be differences in the predictors. Other data from this ongoing study might provide more clarity on this topic. Overall, the results of this study should be interpreted with caution.

### Implications for research and practice

Our findings implicate that already at the beginning of medical school, measures to increase the use of functional coping are needed. Promising starting points are the predictors efficacy, satisfaction with life and emotional distancing. A curricular focus on manageable educational content and measures to improve learning strategies, time management and distancing skills could foster the use of functional coping strategies. Nevertheless, our cross-sectional findings require further longitudinal research.

The present study is limited to the theoretical portion of medical school. In the following clinical year, students face new challenges, including long working hours, insufficient teaching and concerns about medical errors.^53^ Information about the coping behaviour during this phase is needed to identify starting points to foster functional coping strategies not only at this stage of medical school, but also with the aim of a long-term impact on professional life.

## Conclusions

Our cross-sectional findings show differences in the use of functional and dysfunctional coping before beginning medical school and after the first and the fifth years of study. The lower scores for both coping styles after the first year of study require further explanation. The next research step should be verification of our findings with longitudinal data. Efficacy, emotional distancing and satisfaction with life proved to be predictors of functional coping for students after their fifth year. Whether these predictors are adequate starting points to foster functional coping, in particular in the first year of study, remains to be proved.

### Acknowledgements

The authors thank all the study participants. Special thanks are due to Jessica Lückert for her help with statistical analysis and data collection.

### Conflict of Interest

The authors declare that they have no conflict of interest.
